# ﻿Two new species and two new combinations within *Paleosepharia* (Coleoptera, Chrysomelidae, Galerucinae) from China

**DOI:** 10.3897/zookeys.1226.140239

**Published:** 2025-02-06

**Authors:** Chuan Feng, Xingke Yang, Siqin Ge

**Affiliations:** 1 Key Laboratory of Zoological Systematics and Evolution, Institute of Zoology, Chinese Academy of Sciences, 1 Beichen West Road, Chaoyang District, Beijing 100101, China Chinese Academy of Sciences Beijing China; 2 University of Chinese Academy of Sciences, No. 19(A) Yuquan Road, Shijingshan District, Beijing, 100049, China University of Chinese Academy of Sciences Beijing China

**Keywords:** China, identification key, leaf beetles, new record, new combination, taxonomy

## Abstract

In this study, two new species of the leaf-beetle genus *Paleosepharia* Laboissière, 1936 from China are described: *P.subrubra* Feng, Yang & Ge, **sp. nov.** and *P.emeiensis* Feng, Yang & Ge, **sp. nov.** Meanwhile, *Paleosephariatruncata* Laboissière, 1936 is first recorded for China. Additionally, *Monoleptabicavipennis* Chen, 1942 and *Monoleptaquadricavata* Chen, 1976 are redescribed and proposed as new combinations under *Paleosepharia*. A key to the Chinese species of *Paleosepharia* is also provided.

## ﻿Introduction

The subfamily Galerucinae includes three supertribes: Alticitae, Galerucitae, and Serraticollitae (Bezděk and Sekerka 2024). Galerucitae is one of the most diverse groups of leaf beetles, encompassing 7145 species across 543 genera worldwide (Nie et al. 2017). There are 127 genera and 1208 species recorded in China (Yang et al. 2015; Beenen 2024). Among the Galerucitae, the “Monoleptites” is highly diverse, characterized by distinctly elongated first tarsomere of the hind legs. The section “Monoleptites” is a suprageneric, non-monophyletic name and a rank that was established by Chapuis (1875); it is generally accepted to describe a highly diverse group of Galerucitae in the tribe Luperini.

Wilcox’s (1973, 1975) catalogue reports 36 genera of ‘Monoleptites’ worldwide. After revisions by many scholars, such as Wagner (1999, 2003, 2004, 2007, 2011, 2017), Hazmi and Wagner (2010a, 2010b, 2013), Lee (2020), many new genera were established. So far, “Monoleptites” includes approximately 60 genera, with 10 of the genera reported in China (Beenen 2010, 2024), namely: *Atrachya* Chevrolat, 1836, *Chinochya* Lee, 2020, *Macrima* Baly, 1878, *Monolepta* Chevrolat, 1836, *Neochya* Lee, 2020, *Ochralepta* Beenen, 2024, *Paleosepharia* Laboissière, 1936, *Pseudosepharia* Laboissière, 1936, *Trichosepharia* Laboissière, 1936 and *Tsouchya* Lee, 2020. Among these genera, *Monolepta* is the largest (Wagner 2007), with 723 described species worldwide (J. Bezděk 2024, pers. comm.). Apart from *Monolepta*, *Paleosepharia* is the most diverse genus, exhibiting various structural modifications on the elytra (Medvedev 2009, 2012, 2013, 2014; Mohamedsaid 1996, 1999, 2000, 2001).

## ﻿Material and methods

Morphological characters were examined using a Leica S8AP0 microscope.

### ﻿Dissections

The male genitalia of each species were dissected using the following procedure: for dried or ethanol preserved specimens, the abdomen was carefully removed from each specimen, bathed in boiling water for 5–10 minutes, and then transferred to a vial containing 10% KOH solution and bathed for 3–5 min. The abdomen with the aedeagus was washed in distilled water 3 to 4 times, then transferred onto a cavity slide using fine forceps and the aedeagus was separated from the abdomen using a hooked, fine dissecting needle.

### ﻿Photographs

The genitalia were mounted in a drop of glycerol on slides for photography. Images of the habitus and male genitalia were taken using a Canon EOS R5 digital camera. To obtain the full depth of focus, all images were stacked using HELICON FOCUS ver. 7.7.4 and the resulting output was edited with Adobe Photoshop CC 2018.

Labels written in Chinese are translated into English and cited verbatim.

### ﻿Abbreviation used in the paper

**TL** type locality.

**TD** type deposition.

The materials in this study are deposited in the following institutions:

**IZCAS**Institute of Zoology, Chinese Academy of Sciences, Beijing, CHINA

**IZGAS** Institute of Zoology, Guangdong Academy of Sciences, Guangzhou, CHINA

**MNHN**Muséum national d’Histoire naturelle, Paris, FRANCE

**ZMH**Zoological Museum Hamburg, Hamburg, GERMANY

## ﻿Results

### ﻿ Paleosepharia

Taxon classificationAnimaliaColeopteraChrysomelidae

Laboissière, 1936: 251

3A82BCD0-504A-5284-A9AF-94CDCC333344

#### Type species.

*Paleosephariatruncata* Laboissière, 1936, by monotypy and original designation. Redescription in Rizki et al. 2016.

#### Distribution.

Oriental and Palaearctic regions.

#### Diagnosis.

This genus is similar to *Atrachya* Chevrolat, 1836 and *Monolepta* Chevrolat, 1836. In *Paleosepharia*, the third antennomere is longer than the second (both antennomeres are more or less equal in *Monolepta*), and the epipleuron continues towards the apex (suddenly narrowed before the middle in *Monolepta*), procoxal cavity is closed behind (opened in *Atrachya*).

Male genitalia. In *Paleosepharia* and *Atrachya* the aedeagus is parallel-sided in the basal two-thirds, apical third strongly narrowed (aedeagus slender, parallel-sided in *Monolepta*). In *Paleosepharia*, tectum long, rounded or truncate at apex (rounded at apex in *Monolepta*; incised at apex, the two apical tips forming strong hooks in *Atrachya*). *Paleosepharia* usually has two pairs of strongly sclerotized spiculae (lateral and median), while the ventral spiculae are weakly sclerotized. The median spiculae consist of long, slender spikes.

### ﻿ Paleosepharia subrubra

Taxon classificationAnimaliaColeopteraChrysomelidae

Feng, Yang & Ge sp. nov.

67D6D9A7-7AEF-557F-9863-D8152F60573C

https://zoobank.org/9B6BA567-011C-423C-96DC-FF829B69C52E

Figs 1 , 2

#### Type material.

***Holotype***: • ♂, China, Guangdong Province, Chebaling National National Nature Reserve, Xiba, 26 Jul. 2022, Meiying Lin et al. leg., IZGAS.

**Figure 1.**
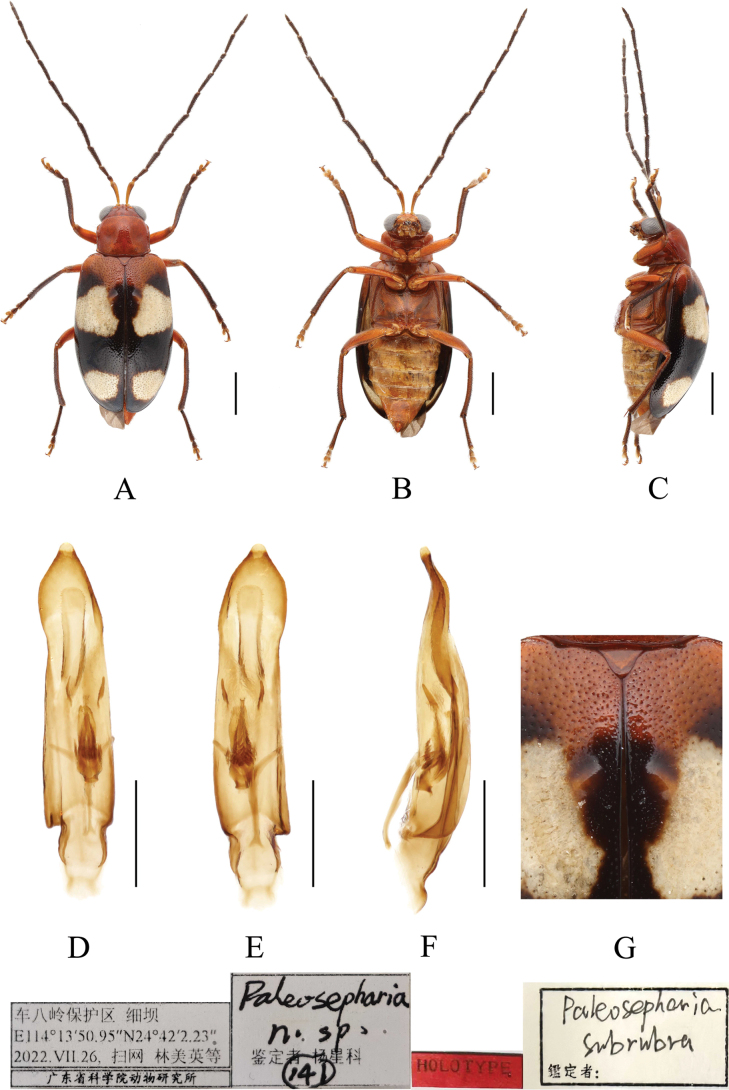
*Paleosephariasubrubra* sp. nov. (holotype, male) **A–C** habitus **D–F** aedeagus **G** depression of the elytra of the male. **A, D** dorsal view **B, E** ventral view **C, F** lateral view. Scale bars: 1 mm (**A–C**); 0.5 mm (**D–F**).

***Paratype***: 1 ♀, • China, Fujian Province, Mount Wuyi, Huangxizhou, 1 Aug. 1997, Youwei Zhang leg., IZCAS.

**Figure 2.**
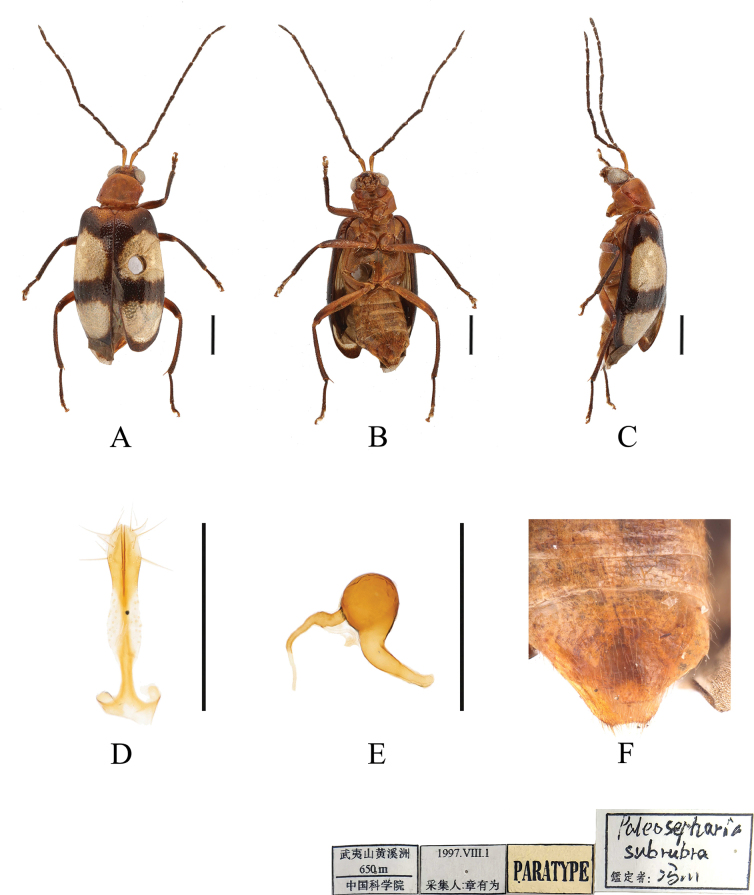
*Paleosephariasubrubra* sp. nov. (paratype, female) **A–C** habitus **D** vaginal palps **E** spermatheca **F** apex of abdomen of the female. **A** dorsal view **B** ventral view **C** lateral view. Scale bars: 1 mm (**A–C**); 0.5 mm (**D**).

#### Etymology.

This species name refers to the red base of the elytra.

#### Diagnosis.

The new species closely resembles *P.kubani* Medvedev, 2004 with both species having black elytra and each elytron with two yellow spots. It differs from *P.kubani* in the elytra with a longitudinal depression at the base and connected to a semicircular depression at the end. In *P.subrubra* sp. nov., the elytra have a rectangular depression at the base. Aedeagus slender, rounded at apex, tectum long, truncate at apex, not reaching apex of the aedeagus.

#### Description.

**Male**: Length: 5.2 mm, width: 2.5 mm. Head, pronotum, scutellum, ventral surface of body and femur reddish-brown, antennae black-brown, with first antennomere reddish-brown. Elytra black and reddish-brown at base, each elytron has two white spots, tibiae and tarsi black. Vertex with sparse punctures. Frontal tubercles transverse, extending downward between antennal bases. Antennae slender, longer than body. First antennomere shiny, bare, rod-shaped, second to eleventh antennomeres with short hairs, second antennomere shortest, third antennomere about 1.55× as long as second; fourth antennomere about 1.78× as long as third, fifth antennomere about 1.1× as long as fourth, sixth to eleventh antennomeres equal in length to fifth. Pronotum about 1.29× as wide as long, lateral margins straight, basal border slightly convex, apical border slightly concave, disc strongly convex, with lateral fovea and dense minute punctures. Scutellum triangular, smooth, impunctate. Elytra wider than pronotum, humeri convex, with pair of weak depressions and pair of rounded protrusions near suture at basal 1/3; disc with dense, minute punctures. Elytral epipleuron broad at base, gradually narrowed from middle to apex. Each tibia with distinct spur at apex, segment 1 of hind tarsi longer than remaining segments combined. Aedeagus slender, with parallel-sided and rounded tip, moderately recurved at apex in lateral view. Lateral spiculae short and slightly curved, ventral spiculae comb-shaped, and median spiculae long.

**Female**: Length: 4.6 mm, width: 2.2 mm.

Third antennomere about 1.8× as long as second; fourth antennomere about 1.6× as long as third. Elytra without protrusions and with slight depressions at base. Spermatheca with big and rounded nodulus, middle part short, and cornu straight. Vaginal palps with wide base and rounded apex, each palp slightly narrowing posteriorly, with 5 setae placed at apex, additional 2 setae subapically.

#### Distribution.

China: Fujian, Guangdong.

### ﻿ Paleosepharia emeiensis

Taxon classificationAnimaliaColeopteraChrysomelidae

Feng, Yang & Ge sp. nov.

1B94D1A8-DB6F-5996-B821-FCDC1B70A6FB

https://zoobank.org/641C30A4-B79B-45AF-9742-7ABDA2AF9487

Figs 3 , 4

#### Type material.

***Holotype***: • ♂, China, Sichuan Province, Mt Emei, Baoguosi, 550–750 m a. s. l., 9 May 1957, Keren Huang leg., IZCAS. ***Paratype***: • 3 ♂♂ 4 ♀♀, same data as for holotype; • 3 ♂♂ 2 ♀♀, China, Sichuan Province, Mt Emei, Baoguosi, 550–750 m a. s. l., 2 Jun. 1957, Keren Huang leg., IZCAS; • 3 ♂♂ 4 ♀♀, China, Sichuan Province, Fengdu County, Shiping, 610 m a. s. l., 3 Jun. 1994, Xingke Yang leg., IZCAS; • 2 ♂♂ 5 ♀♀, China, Sichuan Province, Fengdu County, Shiping, 610 m a. s. l., 3 Jun. 1994, Youwei Zhang leg., IZCAS; • 2 ♂♂ 6 ♀♀, China, Sichuan Province, Fengdu County, Shiping, 610 m a. s. l., 3 Jun. 1994, Wenzhu Li leg., IZCAS.

**Figure 3.**
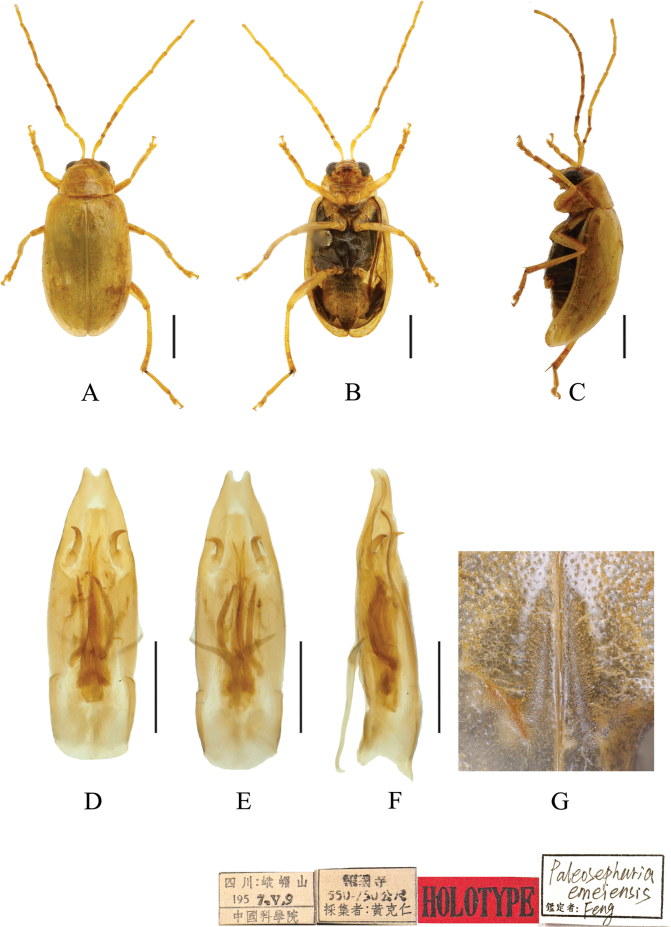
*Paleosephariaemeiensis* sp. nov. (holotype, male) **A–C** habitus **D–F** aedeagus **G** depression of the elytra of the male. **A, D** dorsal view **B, E** ventral view **C, F** lateral view. Scale bars: 1 mm (**A–C**); 0.5 mm (**D–F**).

#### Etymology.

This species name refers to the type locality.

**Figure 4.**
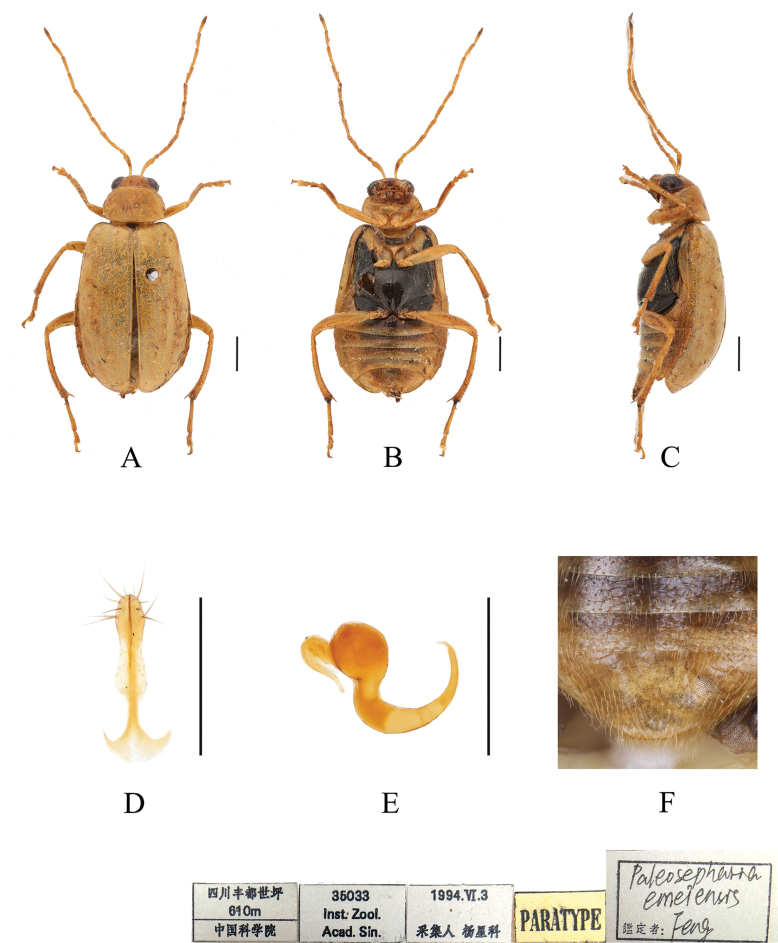
*Paleosephariaemeiensis* sp. nov. (paratype, female) **A–C** habitus **D** vaginal palps **E** spermatheca **F** apex of abdomen of the female. **A** dorsal view **B** ventral view **C** lateral view. Scale bars: 1 mm (**A–C**); 0.5 mm (**D, E**).

#### Diagnosis.

The new species closely resembles *P.fulvicornis* Chen, 1942. It differs from *P.fulvicornis* in the elytra with a longitudinal depression at base, aedeagus slender. In *P.emeiensis*, elytra have a hook-shaped depression at base, and aedeagus robust, 3.1 times longer than wide, parallel-sided in the basal two-thirds, apical third strongly narrowed, apical protrusions nipple-shaped. Tectum long, not reaching the apex of the aedeagus, and truncate at apex.

#### Description.

**Male**: Length: 4.4–5.6 mm, width: 2.2–2.8 mm (mean length 5.2 mm, mean width 2.5 mm, *N* = 7).

General color yellow or yellowish-brown, basal part of inner margin of epipleuron, mesothorax, except mesoepisternum, metathorax and base of first metatarsomere black.

Vertex with sparse punctures. Frontal tubercles transverse, extending downward between antenna bases. Antennae slender, 0.9× as long as body. First antennomere shiny bare, rod-shaped, second to eleventh antennomeres with short hairs, second antennomere shortest, third antennomere 1.8× as long as second, fourth antennomere about 1.3× as long as third, fifth to eleventh antennomeres shorter than fourth and gradually shortened. Pronotum about 1.6× as wide as long, lateral margins slightly curved, basal border slightly convex, apical border slightly concave, disc strongly convex, with dense punctures. Scutellum triangular, smooth, impunctate. Elytra wider than pronotum, humeri convex, disc with dense, minute punctures; and hook-shaped depression behind scutellum. Elytral epipleuron broad at base, continues towards apex. Each tibia with distinct spur at apex, segment 1 of hind tarsi longer than remaining segments combined. Aedeagus robust, with sides slightly rounded, slightly curved at apex in lateral view. Apical protrusions nipple-shaped, small, well separated from each other. Median spiculae with two pairs of slender sclerites, lateral spiculae strongly curved.

**Female**: Length: 4.8–5.8 mm, width: 2.3–3.2 mm (mean length 5.4 mm, mean width 2.6 mm, *N* = 8).

General color yellow or yellowish-brown, scutellum, basal part of inner margin of epipleuron, mesothorax, except mesoepisternum, metathorax and base of first metatarsomere black. Third antennomere about twice as long as second. Elytra lacking longitudinal impression. Spermatheca with big and rounded nodulus, middle part short, and cornu strongly curved. Vaginal palps with wide base and rounded apex, each palp slightly narrowing posteriorly, with 5 setae placed at apex, additional 2 setae subapically.

#### Distribution.

China: Sichuan.

### ﻿ Paleosepharia bicavipennis

Taxon classificationAnimaliaColeopteraChrysomelidae

(Chen, 1942) comb. nov.

7F96DC36-E5D0-5B83-872F-F61079C08F33

Figs 5 , 6 , 7

Monolepta bicavipennis Chen, 1942: 55. TL China: Shaanxi. TDIZCAS.

#### Type specimens examined.

***Holotype***: • ♂ Shensi, Wei-cze-ping, 18 Aug. 1916, P. Licent. ***Paratypes***: • 1 ♂ 2 ♀♀, China, Zhejiang Province, Mount Tianmu, 26 Aug. 1947, IZCAS; • 2 ♀♀, China, Zhejiang Province, Mount Tianmu, 31 Aug. 1947, IZCAS.

**Figure 5.**
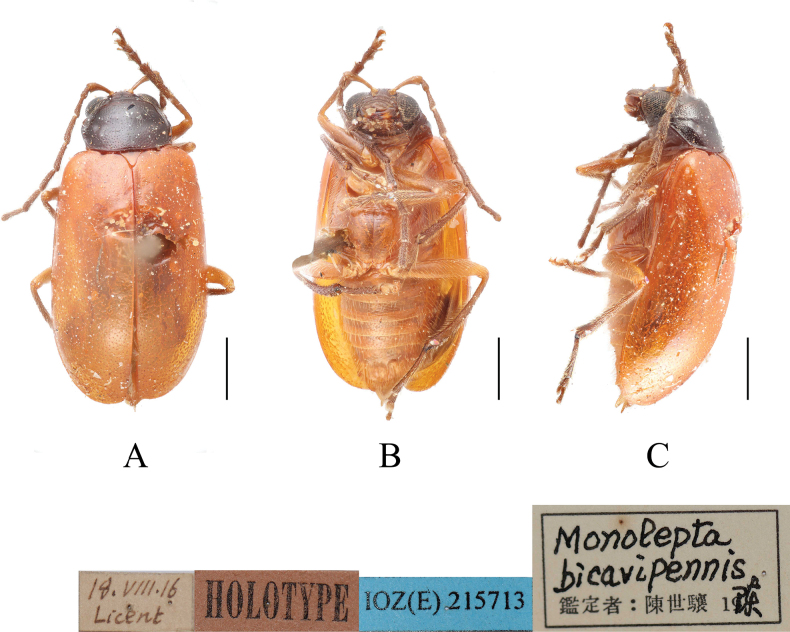
*Paleosephariabicavipennis* (Chen, 1942), comb. nov. (holotype; male) **A–C** habitus. **A** dorsal view **B** ventral view **C** lateral view. Scale bar: 1 mm.

#### Other specimens examined.

7 ♂♂, • China, Henan Province, Song County, Mount Baiyun, 1600 m a. s. l., 19 Jul. 2002, Lijie Zhang leg., IZCAS; • 1 ♂ 5 ♀♀, China, Shaanxi Province, Foping, Yaohe,870–1000 m a. s. l., 25 Jul. 1998, Jun Chen leg., IZCAS; • 5 ♂♂ 1 ♀, China, Gansu Province, Wen County, Bifenggou, 940–1500 m a. s. l., 28 Jul. 1999, Jian Yao leg., IZCAS; • 10 ♂♂ 1 ♀, China, Hubei Province, Shennongjia, Songbai, 950 m a. s. l., 18 Jul. 1980, Peiyu Yu leg., IZCAS; • 9 ♀♀, China, Hunan Province, Yongshun, 600–820 m a. s. l., 7 Aug. 1988, Shuyong Wang leg., IZCAS; • 5 ♂♂ 1 ♀, China, Yunnan Province, Weixi County, Baijixun, 1780 m a. s. l., 10 Jul. 1981, Shuyong Wang leg., IZCAS.

**Figure 6.**
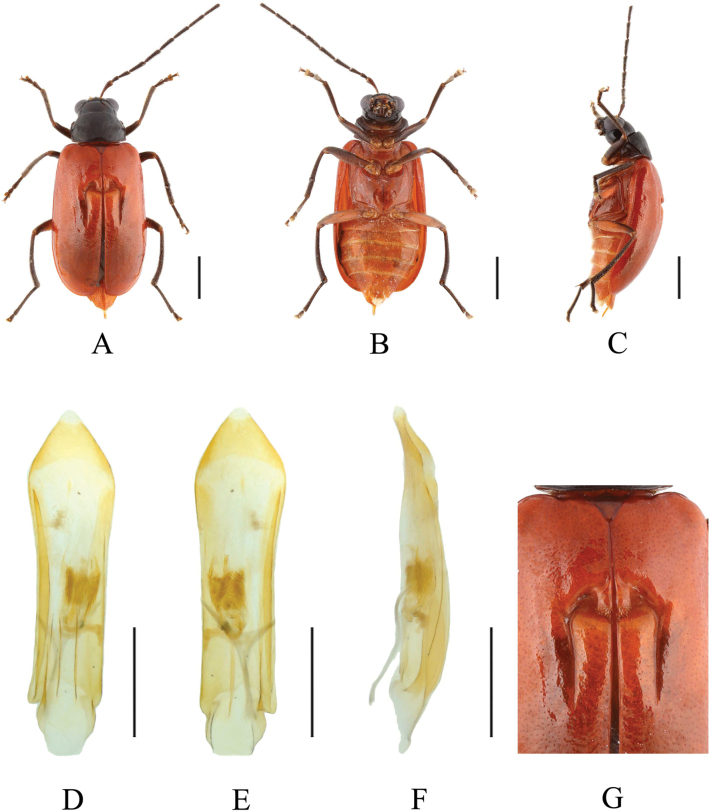
*Paleosephariabicavipennis* (Chen, 1942), comb. nov. (male) **A–C** habitus **D–F** aedeagus **G** depression of the elytra of the male. **A, D** dorsal view **B, E** ventral view **C, F** lateral view. Scale bars: 1 mm (**A–C**); 0.5 mm (**D–F**).

#### Notes.

Based on the following characters of this species, which are characteristic for *Paleosepharia*, it is transferred from *Monolepta* to *Paleosepharia*: the third antennomere is longer than the second; depression in the elytra; median lobe parallel-sided in the basal two-thirds, apical third strongly narrowed; and tectum long, truncate at apex.

**Figure 7.**
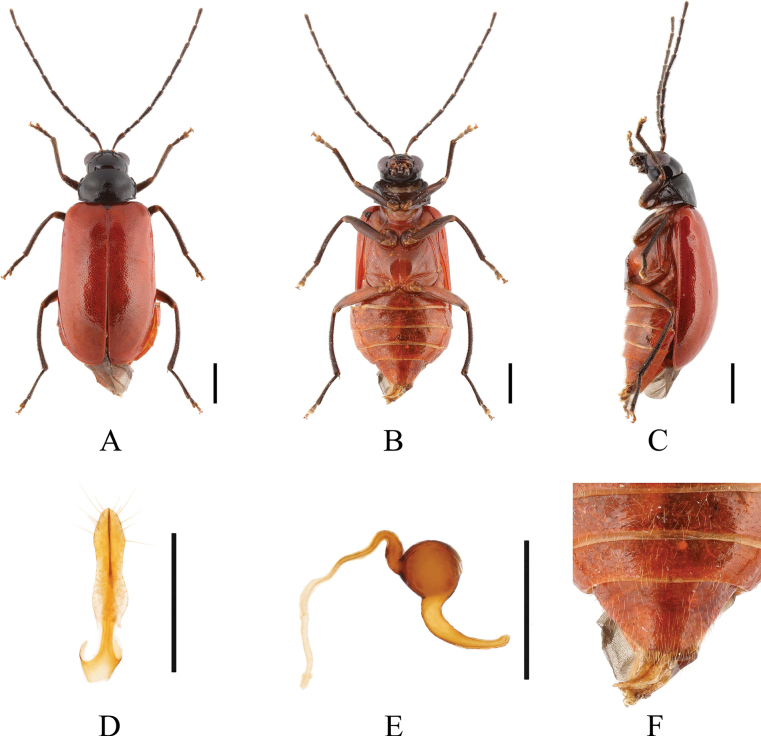
*Paleosephariabicavipennis* (Chen, 1942), comb. nov. (female) **A–C** habitus **D** vaginal palps **E** spermatheca **F** apex of abdomen of the female. **A** dorsal view **B** ventral view **C** lateral view. Scale bars: 1 mm (**A–C**); 0.5 mm (**D, E**).

#### Diagnosis.

This species is easy to distinguish from other species. The elytra is red or yellow-brown, with two comma-shaped deep concavities and two cylindrical protrusions in the middle, and cylindrical protrusions with short hairs.

#### Redescription.

**Male**: Length: 4.2–4.9 mm, width: 2.2–2.6 mm (mean length 4.6 mm, mean width 2.4 mm, *N* = 30). General color yellow-brown or red, head and pronotum black, antennae brown, legs dark brown, ventral side of hind femora yellow-brown, in some specimens, legs largely brown with most tibiae darkened. Vertex with sparse punctures. Frontal tubercles transverse, extending downward between antenna bases. Antennae slender, 0.95× as long as body. First antennomere shiny bare, rod-shaped, second to eleventh antennomeres with short hairs, second antennomere shortest, third antennomere about 1.8× as long as second; fourth antennomere about 1.5× as long as third, fifth to eleventh antennomeres equal in length to fourth. Pronotum about 1.6× as wide as long, lateral margins slightly curved, basal border slightly convex, apical border slightly concave, disc strongly convex, with lateral fovea and dense minute punctures. Scutellum triangular, smooth, impunctate. Elytra wider than pronotum, humeri convex. Each elytron with comma-shaped deep concavity and cylindrical protrusions in middle, cylindrical protrusions with short hairs. In some specimens, longitudinal depression end of concavity elongated or shortened, Disc with dense, minute punctures. Elytral epipleuron broad at base, gradually narrowed from middle to apex. Each tibia with distinct spur at apex, segment 1 of hind tarsi longer than remaining segments combined. Aedeagus robust, gradually widened towards subapex, triangle at apex, moderately recurved at apex in lateral view. Lateral spiculae short, ventral spiculae comb-shaped, and median spiculae long.

**Female**: Length: 4.2–5.0 mm, width: 2.0–2.6 mm (mean length 4.7 mm, mean width 2.4 mm, ***N*** = 21).

Third antennomere about 1.5× as long as second; fourth antennomere about 1.6× as long as third. Elytra without short hairs and depressions. Spermatheca with big and rounded nodulus, middle part short, and cornu slightly curved. Vaginal palps with wide base, anteriorly with subtriangular tip, each palp slightly narrowing posteriorly, with pointed apex, 5 setae placed at apex, additional 2 setae subapically.

#### Distribution.

China: Gansu, Shanxi, Shaanxi, Henan, Anhui, Zhejiang, Hubei, Jiangxi, Hunan, Guangxi, Guizhou, Yunnan.

### ﻿ Paleosepharia quadricavata

Taxon classificationAnimaliaColeopteraChrysomelidae

(Chen et al., 1976) comb. nov.

0D570786-B528-51CD-B328-9AEFAF984C0B

Figs 8 , 9 , 10

Monolepta quadricavata Chen, 1976: 205. TL: China, Xizang; TD: IZCAS.

#### Type material.

***Holotype***: • ♂, China, Xizang Province, Chayu, Shama, 1600 m a. s. l., 20 Jul. 1973, Fusheng Huang leg. IZCAS.

**Figure 8.**
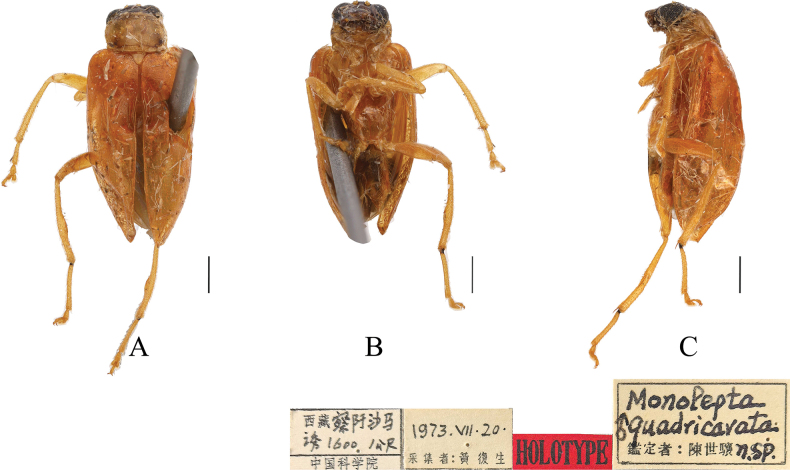
*Paleosephariaquadricavata* (Chen et al., 1976), comb. nov. (holotype, male) **A–C** habitus. **A** dorsal view **B** ventral view **C** lateral view. Scale bar: 1 mm.

#### Other specimens examined.

• 1 ♂, China, Yunan Province, Nujiang, Fugong County, Lumadong, 1200 m a. s. l., 11 Aug. 2024, Chuan Feng leg. IZCAS; • 2 ♀♀, Yunan Province, Fugong County, Maji, Majimi, 1505 m a. s. l., 26 Aug. 2005, Hongbin Liang leg. IZCAS; • 1 ♂, China, Xizang Province, Motuo County, Beibeng, Badeng, 1410 m a. s. l., 24 Jul. 2024, Chuan Feng leg. IZCAS; • 2 ♂♂, China, Xizang Province, Bomi County, Yigong, 2050 m a. s. l., 8 Aug. 2024, Chuan Feng leg. IZCAS; • 4 ♂♂, China, Xizang Province, Motuo County, Beibeng, 799 m a. s. l., 19 Aug. 2015, Jian Yao leg. IZCAS; • 5 ♂♂, China, Xizang Province, Motuo County, Beibeng, 799 m a. s. l., 19 Aug. 2015, Hongbin Liang & Zhengzhong Huang leg. IZCAS; • 5 ♂♂, China, Xizang Province, Motuo County, Ximohe, 707 m a. s. l., 17 Aug. 2015, Jian Yao leg. IZCAS; • 1 ♀, China, Xizang Province, Motuo County, 900 m a. s. l., 27 Aug. 1982, Yinheng Han leg. IZCAS.

**Figure 9.**
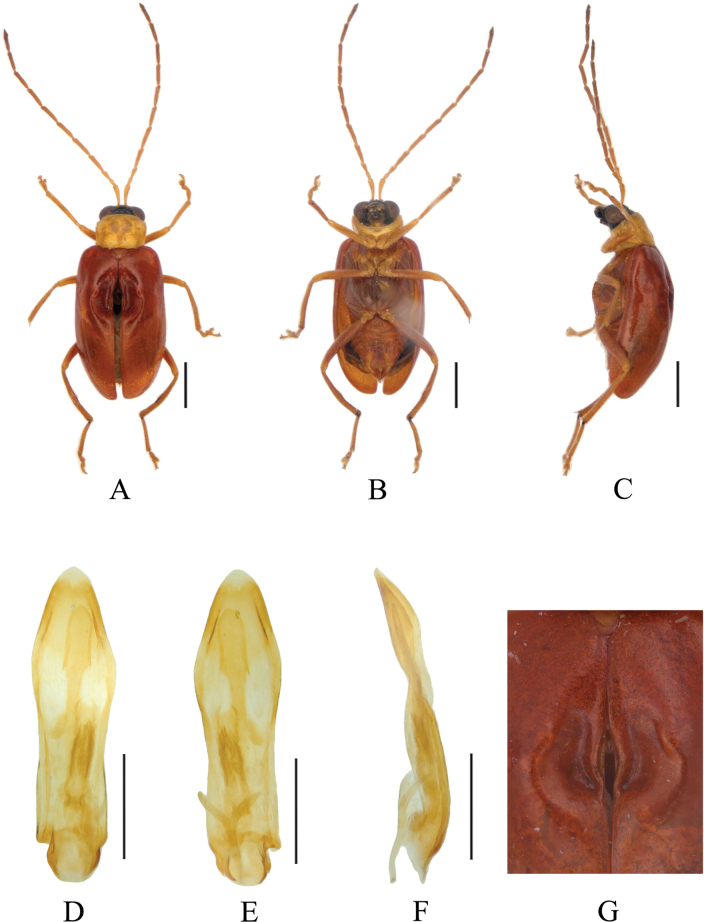
*Paleosephariaquadricavata* (Chen et al., 1976), comb. nov. (male) **A–C** habitus **D–F** aedeagus **G** depression of the elytra of the male. **A, D** dorsal view **B, E** ventral view **C, F** lateral view. Scale bars: 1 mm (**A–C**); 0.5 mm (**D–F**).

#### Notes.

The species is transferred from *Monolepta* to *Paleosepharia* based on the following characters: the third antennomere longer than the second; depression in the elytra; aedeagus parallel-sided in the basal two-thirds, apical third strongly narrowed; tectum long, rounded at apex.

**Figure 10.**
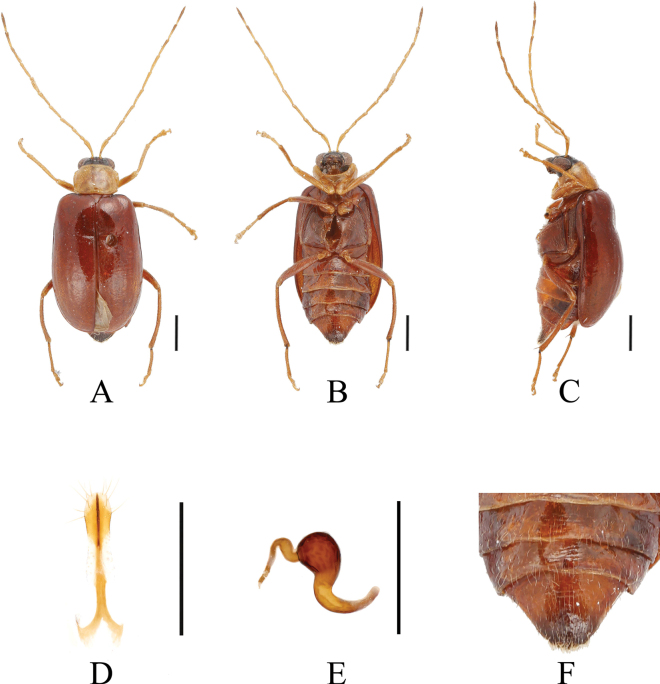
*Paleosephariaquadricavata* (Chen et al., 1976), comb. nov. (female) **A–C** habitus **D** vaginal palps **E** spermatheca **F** apex of abdomen of the female. **A** dorsal view **B** ventral view **C** lateral view. Scale bars: 1 mm (**A–C**); 0.5 mm (**D, E**).

#### Diagnosis.

This species is easy to distinguish from other species based on the elytra red or reddish-brown, with a large, deep concavity near suture in male.

#### Redescription.

**Male**: Length: 4.2–5.0 mm, width: 2.0–2.4 mm (mean length 4.6 mm, mean width 2.1 mm, *N* = 19).

General color red or reddish-brown, head black, pronotum yellow, antennae and legs brown.

Vertex with sparse punctures. Frontal tubercles transverse, extending downward between antennal bases. Antennae slender, equal to length of body. First antennomere shiny bare, rod-shaped, second to eleventh antennomeres with short hairs, second antennomere shortest, third antennomere about 1.3× as long as second; fourth antennomere about twice as long as third, fifth antennomere about 1.2× as long as fourth, sixth to eleventh antennomeres equal in length to fourth. Pronotum about 1.4× as wide as long, lateral margins straight, basal border slightly concaved in middle, apical border straight, disc with lateral fovea and dense minute punctures. Scutellum triangular, smooth, impunctate. Elytra wider than pronotum, humeri convex, with large, deep concavity near suture at basal 1/3, disc with dense, minute punctures. Elytral epipleuron broad at base, gradually narrowed from middle to apex. Each tibia with distinct spur at apex, segment 1 of hind tarsi longer than remaining segments combined. Aedeagus robust, close to outsole shape in dorsal and ventral views, moderately recurved at apex in lateral view. Lateral spiculae short and curved, ventral spiculae comb-shaped, and median spiculae long.

**Female**: Length: 4.2–5.0 mm, width: 2.1–2.6 mm (mean length 4.7 mm, mean width 2.3 mm, ***N*** = 3).

Third antennomere about 1.6× as long as second; fourth antennomere about 1.6× as long as third. Elytra only with one slight depression at basal 1/3. Spermatheca with big and rounded nodulus, middle part short, cornu short and slightly curved. Vaginal palps with wide base and rounded apex, each palp slightly narrowing posteriorly, with 5 setae placed at apex, additional 2 setae subapically.

#### Distribution.

China: Yunnan, Xizang; Nepal.

##### ﻿New record

### ﻿ Paleosepharia truncata

Taxon classificationAnimaliaColeopteraChrysomelidae

Laboissière, 1936

B27B66F2-12A0-5BF4-A53F-CC4EAB8716F4

Figs 11 , 12 , 13

Paleosepharia truncata Laboissière 1936: 251. TL Vietnam. TDMNHN, ZMH.

#### Type material.

***Cotype***, • ♂, Le Moult Vend. via Reinbek Eing. Nr. 1, 1957. *Paleosephariatruncata* V. Laboissière Det. MUSEUM PARIS COCHINCHINE RIERRE 1878. ***Paralectotypus****Paleosephariatruncata* Laboissière, 1936 Hazmi et al. desig. ZMH 844689.

**Figure 11.**
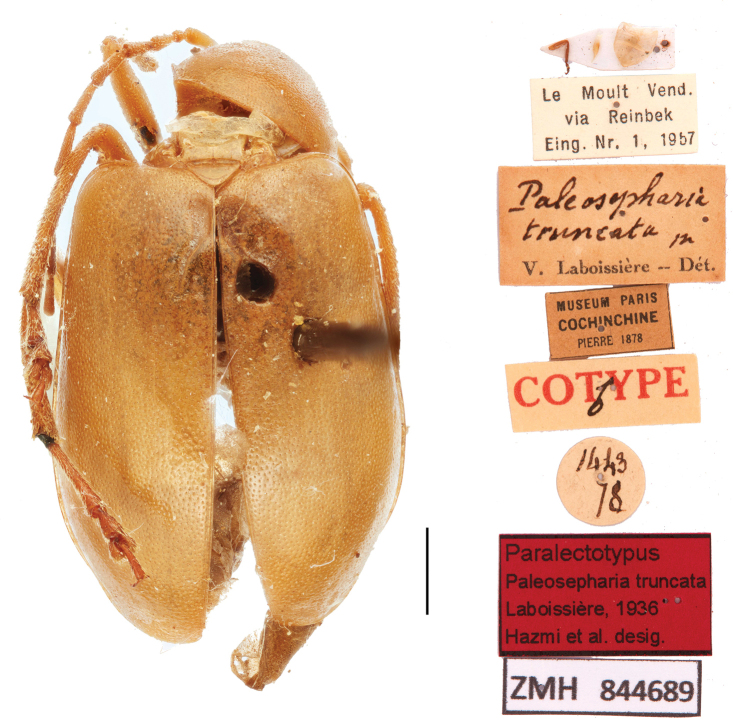
*Paleosephariatruncata* Laboissière, 1936 (paratype). Scale bar: 1 mm.

#### Other specimens examined.

• 1 ♂, China, Yunan Province, Xishuangbanna, Mengzhe, 1700 m a. s. l., 22 Jun. 1958, Shuyong Wang leg., IZCAS; • 1 ♀, China, Yunan Province, Xishuangbanna, Menghun, 1200 m a. s. l., 4 Jun. 1958, Shuyong Wang leg., IZCAS; • 1 ♀, China, Yunan Province, Xishuangbanna, Menga, 1050–1080 m a. s. l., 8 Jun. 1958, Shuyong Wang leg., IZCAS; • 1 ♂, China, Yunan Province, Xishuangbanna, Mengzhe, 1700 m a. s. l., 10 Jun. 1958, Shuyong Wang leg., IZCAS; • 1 ♀, China, Yunan Province, Xishuangbanna, Mengzhe, 1200 m a. s. l., 15 Jun. 1958, Shuyong Wang leg., IZCAS.

**Figure 12.**
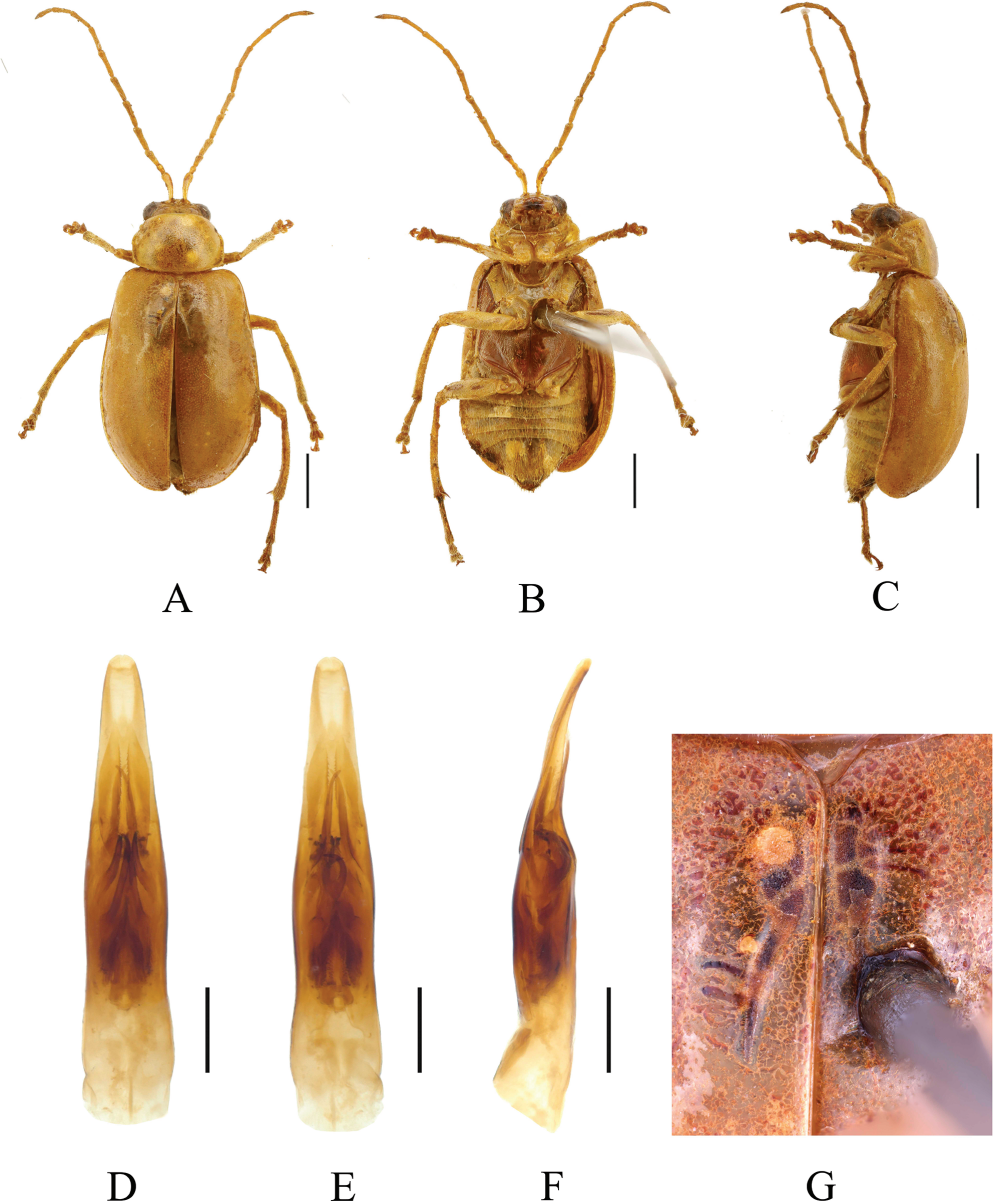
*Paleosephariatruncata* Laboissière, 1936 (male) **A–C** habitus **D–F** aedeagus **G** depression of the elytra of the male. **A, D** dorsal view **B, E** ventral view **C, F** lateral view. Scale bars: 1 mm (**A–C**); 0.5 mm (**D–F**).

#### Description.

**Male**: Length: 6.8 mm, width: 3.8 mm.

**Figure 13.**
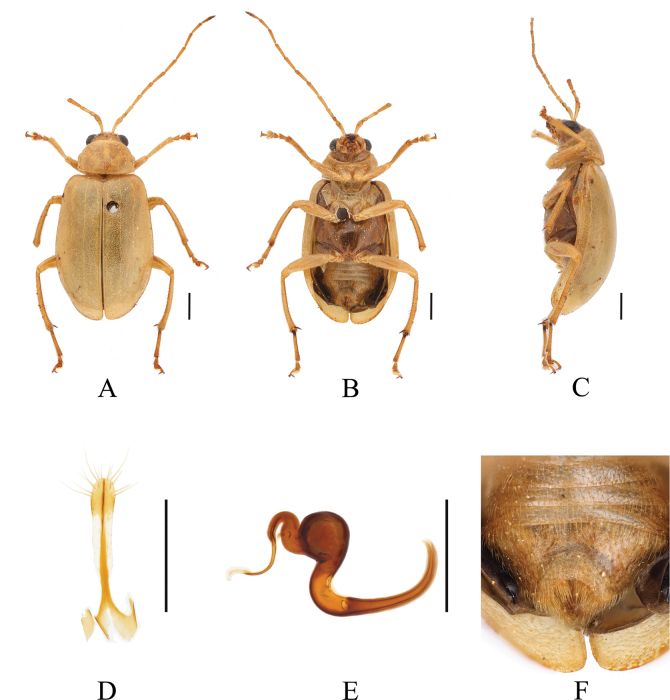
*Paleosephariatruncata* Laboissière, 1936 (female) **A-C** habitus **D** vaginal palps **E** spermatheca **F** apex of abdomen of the female. **A** dorsal view **B** ventral view **C** lateral view. Scale bars: 1 mm (**A–C**); 0.5 mm (**D, E**).

General color yellow or yellowish-brown, metathorax reddish-brown, basal part of inner margin of epipleuron and base of first metatarsomere black.

Vertex with sparse punctures. Frontal tubercles transverse, extending downward between antenna bases. Antennae slender, equal to length of body. First antennomere shiny bare, rod-shaped, second to eleventh antennomeres with short hairs, second antennomere shortest, third antennomere 1.9× as long as second, fourth antennomere about 1.4× as long as third, fifth to seventh antennomeres slightly longer than fourth, eighth to eleventh antennomeres equal in length to fourth. Pronotum about 1.55× as wide as long, lateral margins slightly curved, basal border slightly convex, apical border slightly concave, disc strongly convex, with dense punctures. Scutellum triangular, smooth, impunctate. Elytra wider than pronotum, humeri convex, disc with dense, minute punctures; with elliptical postscutellar depression that extends obliquely backwards. Elytral epipleuron broad at base, continues towards apex. Each tibia with distinct spur at apex, segment 1 of hind tarsi longer than remaining segments combined. Aedeagus gradually narrowed towards apex, slightly curved at apex in lateral view. lateral and median spiculae clearly visible, ventral spiculae weakly. Median spiculae with two pairs of slender sclerites.

**Female**: Length: 7.4–8.2 mm, width: 4.2–4.4 mm (mean length 7.7 mm, mean width 4.3 mm, ***N*** = 4).

Elytra lacking impression. Apex of abdomen with rounded concave. Spermatheca with big and rounded nodulus, middle part short, cornu long and strongly curved. Vaginal palps gradually widened towards apex, each palp with 5 setae placed at apex, additional 2 setae subapically.

#### Distribution.

China: Yunnan; Vietnam, Thailand, Laos.

### ﻿Key to Chinese species of *Paleosepharia*

**Table:** 

1	Elytra unicolored	**2**
–	Elytra bicolor or tricolor	**6**
2	Elytra black	***P.subnigra* Gressitt & Kimoto, 1963**
–	Elytra yellow or yellow-brown or red or reddish-brown	**3**
3	Head black	**4**
–	Head yellow or yellow-brown	**5**
4	Pronotum black, Elytra with comma-shaped deep concavity and cylindrical protrusions in male	***P.bicavipennis* (Chen, 1942)**
–	Pronotum yellow, Elytra with a large, deep concavity near suture at basal 1/3 in male	***P.quadricavata* (Chen, 1976)**
5	Metasternum black	***P.kolthoffi* Laboissière, 1938**
–	Metasternum yellow	***P.pallens* Chen in Wang & Yang, 1998**
6	Elytra bicolor	**7**
–	Elytra tricolor	**31**
7	Elytra with yellow and black	**8**
–	Elytra with yellow and red or yellow-brown and reddish-brown	**27**
8	Elytra without stripes or spots	**9**
–	Elytra with stripes or spots	**18**
9	Basal part of inner margin of epipleuron black	**10**
–	Lateral margins of elytra black	**16**
10	Basal margin of pronotum with a tongue-shaped process in male, slightly process in female	***P.lingulata* Chen & Jiang, 1984**
–	Basal margin of pronotum without any process	**11**
11	Metathorax reddish-brown	**12**
–	Mesothorax black	**13**
12	Abdomen yellow, body length 6.8–8.2 mm	***P.truncata* Laboissière, 1936**
–	Abdomen red, body length 4.5 mm	***P.truncatipennis* Chen & Jiang, 1984**
13	Scutellum black, elytra with spindle-shaped depressions in male	***P.fusiformis* Chen & Jiang, 1984**
–	Scutellum yellow	**14**
14	Elytra with oval depressions in male	***P.orbiculata* Chen & Jiang, 1984**
–	Elytra with hook-shaped depressions in male	**15**
15	Aedeagus robust, 3.1 times longer than wide, and apical protrusions nipple-shaped	***P.emeiensis* sp.nov.**
–	Aedeagus slender, 4 times longer than wide, apex without large protrusions	***P.fulvicornis* Chen, 1942**
16	Vertex black, the depression extending from the scutellum to the middle of the elytra in male	***P.verticalis* Chen & Jiang, 1984**
–	Vertex yellow or yellow-brown	**17**
17	Apical 1/2 of suture black	***P.basituberculata* Chen & Jiang, 1984**
–	Basal 1/2 of suture black	***P.caudata* Chen & Jiang, 1984**
18	Elytra with black stripes	**19**
–	Elytra with yellow stripes or spots	**26**
19	Elytra with one black stripe	**20**
–	Elytra with two black stripes	**24**
20	Elytral transverse stripe before middle disc	***P.amiana* (Chûjô, 1962)**
–	Elytral transverse stripe after middle disc	**21**
21	Elytral stripe at apex	**22**
–	Elytral stripe subapical	***P.liquidambara* Gressitt & Kimoto, 1963**
22	Lateral margins of elytra black	***P.posticata* Chen, 1942**
–	Lateral margins of elytra yellow	23
23	Antennal segment 5 longer than 4, postscutellar depressions straight in male	***P.quercicola* Chen & Jiang, 1984**
–	Antennal segment 5 as long as 4, postscutellar depressions “J”-shaped	***P.j-signata* Chen & Jiang, 1984**
24	Elytral anterior transverse black bands connected	**25**
–	Elytra without anterior transverse black band near the suture	***P.yasumatsui* (Kimoto, 1969)**
25	Elytra with slender transverse black bands and weakly margined	***P.excavata* (Chûjô, 1938)**
–	Elytra with broad transverse black bands and distinctly margined	***P.formosana* (Chûjô, 1935)**
26	Elytra black; with one pair of curved yellow stripes	***P.nantouensis* (Kimoto, 1996)**
–	Elytra black; each elytron with a yellow spot	***P.fasciata* Gressitt & Kimoto, 1963**
27	Elytra red, each elytron with a yellow spot	***P.gongshana* Chen & Jiang, 1986**
–	Elytra yellow or yellow-brown, and only a few red	**28**
28	Humeral angle red	**29**
–	Humeral angle yellow	**30**
29	Basal and apical l/10 of elytra red, outside tibia blackish-brown, elytra without depression	***P.basipennis* Gressitt & Kimoto, 1963**
–	Basal 1/3 of elytral epipleuron red; tibia brown, elytra with slender depression in mela	***P.humeralis* Chen & Jiang, 1984**
30	Elytra with a red stripe after middle disc	***P.jiangae* Beenen, 2008**
–	Elytra without stripe, and basal part of inner margin of epipleuron red	***P.tibialis* Chen & Jiang, 1984**
31	Elytra red at basal and apical l/10, without spot	***P.castanoceps* Chen & Jiang, 1984**
–	Elytra red at base, each elytron with two white spots	***P.subrubra* sp. nov.**

## ﻿Discussion

Currently, there are 32 known species of *Paleosepharia* in China and 87 species in the world. However, in the process of studying this genus, it is found that there are several characteristics that are inconsistent in *Paleosepharia*, such as the presence or absence of a spine at the end of the tibia of the fore leg, the presence or absence of a depression on the male elytra, the width of the epipleuron, the shape of the VIII sternite, etc. If reasonable, future research could explore subdividing *Paleosepharia* into several groups based on a combination of several key features. Moreover, we aim to use more extensive data to verify the monophyly of these groups.

## Supplementary Material

XML Treatment for Paleosepharia

XML Treatment for Paleosepharia subrubra

XML Treatment for Paleosepharia emeiensis

XML Treatment for Paleosepharia bicavipennis

XML Treatment for Paleosepharia quadricavata

XML Treatment for Paleosepharia truncata

## ﻿Additional information

### Author ORCIDs

Chuan Feng https://orcid.org/0009-0001-9299-3526

Xingke Yang https://orcid.org/0000-0002-0870-4657

Siqin Ge https://orcid.org/0000-0001-5924-3400

### Data availability

All of the data that support the findings of this study are available in the main text.
